# Neurosurgical Randomized Trials in Low- and Middle-Income Countries

**DOI:** 10.1093/neuros/nyaa049

**Published:** 2020-03-14

**Authors:** Dylan P Griswold, Ahsan A Khan, Tiffany E Chao, David J Clark, Karol Budohoski, B Indira Devi, Tej D Azad, Gerald A Grant, Rikin A Trivedi, Andres M Rubiano, Walter D Johnson, Kee B Park, Marike Broekman, Franco Servadei, Peter J Hutchinson, Angelos G Kolias

**Affiliations:** Stanford University School of Medicine, Stanford, California; Division of Neurosurgery, Department of Clinical Neurosciences, Addenbrooke's Hospital, University of Cambridge, Cambridge, United Kingdom; NIHR Global Health Research Group on Neurotrauma, University of Cambridge, Cambridge, United Kingdom; Neuroscience Institute, INUB-MEDITECH Research Group, El Bosque University, Bogotá, Colombia; Stanford University School of Medicine, Stanford, California; Department of Surgery, Santa Clara Valley Medical Center, San Jose, California; NIHR Global Health Research Group on Neurotrauma, University of Cambridge, Cambridge, United Kingdom; Division of Neurosurgery, Department of Clinical Neurosciences, Addenbrooke's Hospital, University of Cambridge, Cambridge, United Kingdom; NIHR Global Health Research Group on Neurotrauma, University of Cambridge, Cambridge, United Kingdom; Division of Neurosurgery, Department of Clinical Neurosciences, Addenbrooke's Hospital, University of Cambridge, Cambridge, United Kingdom; NIHR Global Health Research Group on Neurotrauma, University of Cambridge, Cambridge, United Kingdom; Department of Neurosurgery, National Institute of Mental Health and Neurosciences (NIMHANS), Bangalore, India; Stanford University School of Medicine, Stanford, California; Department of Neurosurgery, Johns Hopkins University, Baltimore, Maryland; Stanford University School of Medicine, Stanford, California; NIHR Global Health Research Group on Neurotrauma, University of Cambridge, Cambridge, United Kingdom; Division of Neurosurgery, Department of Clinical Neurosciences, Addenbrooke's Hospital, University of Cambridge, Cambridge, United Kingdom; NIHR Global Health Research Group on Neurotrauma, University of Cambridge, Cambridge, United Kingdom; Neuroscience Institute, INUB-MEDITECH Research Group, El Bosque University, Bogotá, Colombia; Emergency and Essential Surgical Care Programme, World Health Organization, Geneva, Switzerland; Global Neurosurgery Initiative, Program in Global Surgery and Social Change, Department of Global Health and Social Medicine, Harvard Medical School, Boston, Massachusetts; Department of Neurosurgery, Leiden University Medical Center, Leiden and Haaglanden Medical Center, the Hague, the Netherlands; Department of Neurosurgery, Humanitas Research Hospital, Humanitas University, Milan, Italy; NIHR Global Health Research Group on Neurotrauma, University of Cambridge, Cambridge, United Kingdom; Division of Neurosurgery, Department of Clinical Neurosciences, Addenbrooke's Hospital, University of Cambridge, Cambridge, United Kingdom; NIHR Global Health Research Group on Neurotrauma, University of Cambridge, Cambridge, United Kingdom; Division of Neurosurgery, Department of Clinical Neurosciences, Addenbrooke's Hospital, University of Cambridge, Cambridge, United Kingdom

**Keywords:** Neurosurgery, Spinal surgery, Neurotrauma, Research, Global health, Global neurosurgery, Global surgery, Low- and middle-income countries, Research capacity strengthening, Health disparities, Access to care

## Abstract

**BACKGROUND:**

The setting of a randomized trial can determine whether its findings are generalizable and can therefore apply to different settings. The contribution of low- and middle-income countries (LMICs) to neurosurgical randomized trials has not been systematically described before.

**OBJECTIVE:**

To perform a systematic analysis of design characteristics and methodology, funding source, and interventions studied between trials led by and/or conducted in high-income countries (HICs) vs LMICs.

**METHODS:**

From January 2003 to July 2016, English-language trials with >5 patients assessing any one neurosurgical procedure against another procedure, nonsurgical treatment, or no treatment were retrieved from MEDLINE, Scopus, and Cochrane Library. Income classification for each country was assessed using the World Bank Atlas method.

**RESULTS:**

A total of 73.3% of the 397 studies that met inclusion criteria were led by HICs, whereas 26.7% were led by LMICs. Of the 106 LMIC-led studies, 71 were led by China. If China is excluded, only 8.8% were led by LMICs. HIC-led trials enrolled a median of 92 patients vs a median of 65 patients in LMIC-led trials. HIC-led trials enrolled from 7.6 sites vs 1.8 sites in LMIC-led studies. Over half of LMIC-led trials were institutionally funded (54.7%). The majority of both HIC- and LMIC-led trials evaluated spinal neurosurgery, 68% and 71.7%, respectively.

**CONCLUSION:**

We have established that there is a substantial disparity between HICs and LMICs in the number of published neurosurgical trials. A concerted effort to invest in research capacity building in LMICs is an essential step towards ensuring context- and resource-specific high-quality evidence is generated.

**ABBREVIATIONS**
GDP: gross domestic product
GNI: gross national income
GSK: GlaxoSmithKline
HIC: high-income country
IQR: interquartile range
LMIC: low- and middle-income country
RCT: randomized clinical trial
SD: standard deviation
SDG: Sustainable Development Goal
TBI: traumatic brain injury

The availability of neurosurgical services in low- and middle-income countries (LMICs) is limited. In the first study to quantify geographic access to neurosurgical care, Punchak et al^1^ found that individuals living in 11 of the 68 countries providing data reported having no practicing neurosurgeons. The same authors found that the “average percentage of the population with access to neurosurgical services within a 2-h window is 25.26% in Sub-Saharan Africa, 62.3% in Latin America and the Caribbean, 29.64% in East Asia and the Pacific, 52.83% in South Asia, 79.65% in the Middle East and North Africa, and 93.3% in Eastern Europe and Central Asia.”^1^ When access to both basic neurosurgery and advanced microneurosurgery is evaluated, the percentage of the population with access to higher levels of neurosurgical care decreases even further. Therefore, with such a divergence in resources and capacity, it becomes increasingly important to develop best-practice evidence-based guidelines using these sample populations. Furthermore, because those working and living in LMICs are better placed to define issues of importance to their populations than are people living thousands of miles away in high-income countries (HICs), institutes and researchers in LMICs are best equipped to conduct research to find local solutions to local problems.^2^

In the era of evidence-based medicine, in which there is an increasing focus on the implementation of best-practice guidelines, it is important to remember that research studies are carried out on a particular sample of subjects and that results of a particular study may not be generalizable to distinct populations.^3^ This becomes increasingly important in the development of best-practice guidelines for LMICs. Given the substantial differences in health resources, infrastructure, technology, medical personnel, and environmental factors that exist between HICs and LMICs, making decisions about healthcare delivery in LMICs likely requires a different evidence base, at least to some extent. For areas of research on complex noncommunicable diseases like cancer or in the surgical management of trauma, the weight given to the provision of local resources and the capacity of regional health systems makes extrapolating from research conducted by HICs problematic and can potentially lead to inappropriate conclusions and treatment strategies which are impossible to implement.^4^

Thus, we sought to appraise neurosurgical randomized clinical trials (RCTs) conducted between 2003 and 2016 in order to compare design characteristics and methodology, source of funding, and intervention studied between trials led by and/or conducted in HICs and LMICs. Understanding the gap and difference in neurosurgical research between LMICs and HICs is a critical step in ensuring best-practice, evidence-based guidelines tailored to LMICs are developed appropriately. An evidence-based approach to healthcare provides state and nonstate actors with the tools necessary to guide public health policy decision-making.^5,6^

## METHODS

### Systematic Review and Search Strategy

In an effort to remain consistent with the previous work of Vranos et al^7^ and Azad et al^8^ to characterize and assess the state of neurosurgical RCTs, we utilized the database of RCTs described by Azad et al^8^ for our data extraction. The search strategy and review protocol are outlined in the supplementary data of their report. In brief, Azad et al^8^ searched MEDLINE, Scopus, and the Cochrane Library Controlled Trials Registry from January 2003 to July 2016 aiming to identify randomized trials of neurosurgical procedures used in “cranial and spinal neurosurgical practice, excluding trials of peripheral nerve procedures.”^8^ All randomized trials compared one neurosurgical procedure with another neurosurgical procedure, nonsurgical treatment, or no treatment. Studies with <5 patients in each arm and studies that compared drugs, physical therapy, or conventional radiotherapy alone were excluded. The exclusion strategy of Vranos et al^7^ was used, in which trials generally performed by non-neurosurgeons were excluded.

### Data Extraction

The database was previously existing and provided by authors T.D.A. and G.A.G. We added variables that were relevant and necessary to compare RCTs between HICs and LMICs. The following data were extracted from each study: publication year, funding source, study size, age group, RCT methodology, indicated subspecialty, intervention, control, primary outcome measure, secondary outcome measures, enrollment period, follow-up duration, follow-up method, lead institution, lead institution country, participating countries, number of trial sites, number of HIC sites, and number of LMIC sites. The lead institution and enrollment site variables were defined according to the first author's institutional affiliation. For the purposes of this study, 2 authors (D.G. and A.A.K.) independently screened all studies included in the database and extracted all necessary details. All studies were reassessed and compared to criteria included in the 2013 SPIRIT statement.^9^ Conflicts were resolved by consensus and/or review by the senior author.

The classification of each country's income level was assessed using the World Bank Atlas method^10^ and sorted according to the 2019 fiscal year data, defining low-income economies as those with a gross national income (GNI) per capita of $995 or less in 2017. Lower-middle, upper-middle, and high-income economies are defined as those with a GNI per capita between $996 and $3895; $3896 and $12,055; and $12,056 or more, respectively.^11^

### Statistical Analysis

We are reporting descriptive statistics with regards to the characteristics of interest. Analyses of categorical data were based on chi-square tests, and analyses of continuous variables were based on Mann-Whitney *U* tests. The geographic coordinate heatmap was generated using open source code provided by Babicki et al.^12^

## RESULTS

**FIGURE 1.**
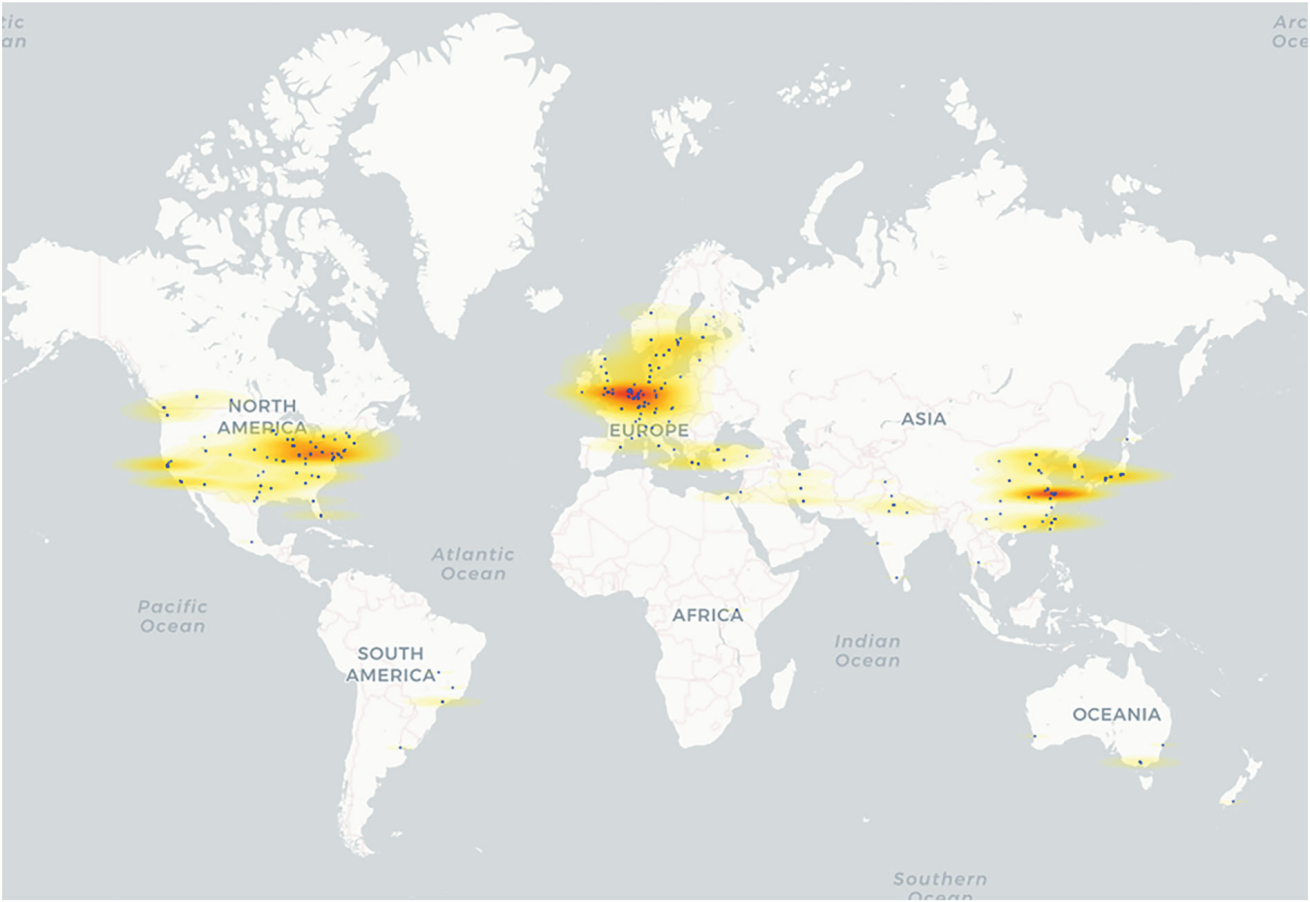
Application programming interface (API) coordinates of lead sites reveals the concentration of hubs of research. Each blue dot represents the location of the lead site. ArcGIS software was used to generate a kernel density heat map based on the latitude and longitude of each site.

A total of 397 studies met the inclusion criteria and were selected for data extraction. Using the first author's institutional affiliation, 55 separate countries were represented throughout the 397 studies as either the lead institute or site of enrollment. Of these 55 countries, 52.8% (n = 32) were HICs, whereas 41.8% (n = 23) were LMICs. Of the 397 studies, 73.3% (n = 291) were led by HICs, whereas 26.7% (n = 106) were led by LMICs. However, of the 106 LMIC-led studies, 71 were led by China. Excluding China, only 8.8% (n = 35) RCTs were led by LMICs. Finally, of the 397 studies, 283 exclusively enrolled patients from HICs, compared to the 108 studies with an exclusively LMIC population. Only 2.0% (n = 6) of studies recruited patients from both HICs and LMICs. The map presented in Figure 1 clearly illustrates the concentration of centers leading neurosurgical RCTs in specific regions. The regions of highest densities include Western Europe, the East Coast of the United States, and the metropolitan areas of China. Most of South America, all of Africa, Central Asia, the Middle East, and South Asia stand in stark contrast to the aforementioned regions.

### Design Characteristics of HIC and LMIC Neurosurgical RCTs

The median sample size for HIC- vs LMIC-led RCTs differed significantly with a median of 92 patients (interquartile range [IQR], 46-182) enrolled in HIC-led studies vs a median of 65 patients (IQR, 42-106) enrolled in LMIC-led studies (*P* = .0086). The median length of enrollment in HIC-led studies was 36 mo (IQR, 21-51) and 30 mo (IQR, 20-47) in LMIC-led studies (*P* = .22). The median length of follow-up in HIC-led studies was 24 mo (IQR, 12-33) and 18 mo (IQR, 12-32) in LMIC-led studies (*P* = .26). HIC-led studies enrolled an average of 7.6 sites (standard deviation [SD], ±12.0) vs an average of 1.8 sites (SD, ±4.7) enrolled in LMIC-led studies (*P* < .0001; Table 1).

**TABLE 1.** Comparison of Randomized Controlled Trials Led by High-Income Countries vs Low- and Middle-Income Countries

Characteristics	HIC-led	LMIC-led	*P* value
Total studies—no./total no. (%)	291/397 (73.3)	106/397 (26.7)	–
Median sample size (IQR)	92 (46-182)	65 (42-106)	.0086
Median length of enrollment (IQR)	36 (21-51)	30 (20-47)	.22
Median length of follow-up (IQR)	24 (12-33)	18 (12-32)	.26
Mean number of sites (SD)	7.6 (12.0)	1.8 (4.7)	<.0001
Source of funding—no./total no. (%)			
Institutional	94/291 (32.3)	58/106 (54.7)	<.0001
Industry	98/291 (33.7)	4/106 (3.8)	
Government	63/291 (21.6)	20/106 (18.9)	
Charitable	9/291 (3.1)	2/106 (1.9)	
Unspecified	27/291 (9.3)	22/106 (20.7)	
Subspecialty—no./total no. (%)			
Spine	198/291 (68.0)	76/106 (71.7)	<.0001
Functional	37/291 (12.7)	3/106 (2.8)	
Cerebrovascular	25/291 (8.6)	12/106 (11.3)	
Neurotrauma	6/291 (2.1)	8/106 (7.5)	
General	11/291 (3.8)	1/106 (0.9)	
Neuro-oncology	10/291 (3.4)	3/106 (2.8)	
Pediatric	4/291 (1.4)	3/106 (2.8)	

### Comparison of Funding Sources in HIC and LMIC Neurosurgical RCTs

A clear difference was found between the origin of funding sources in HIC-led and LMIC-led RCTs (*P* < .0001). Notably, the greatest proportion of funding for HIC-led studies came from industry (33.7%, n = 98) with institutionally funded studies close behind (32.3%, n = 94). In LMIC-led studies, over half were institutionally funded (54.7%, n = 58). Only 3.8% (n = 4) of LMIC-led studies reported receiving funding from industry, 3 of which were led by China. Government funding was similar between both HIC-led studies (21.6%, n = 63) and LMIC-led studies (18.9%, n = 20).

### Comparison of Subspecialties

Across all subspecialties, the distribution of studies differs significantly between HIC-led and LMIC-led RCTs. Studies evaluating spinal neurosurgery were equally as dominant in LMIC-led studies (71.7%, n = 76) as they were in HIC-led studies (68.0%, n = 198). Whereas 12.7% (n = 37) of HIC-led RCTs fell into a functional category, only 2.8% (n = 3) of LMIC-led studies evaluated functional neurosurgery. Only 7.5% (n = 8) of LMIC-led studies focused on neurotrauma.

Similar patterns across all of these comparisons (design characteristics, funding, and subspecialties) emerged when comparing studies with HIC-only participants and LMIC-only participants (Table 2).

**TABLE 2.** Comparison of Neurosurgical Randomized Controlled Trials Sample Populations in High-Income Countries vs Low- and Middle-Income Countries

Characteristics	HIC-only sample	LMIC-only sample	*P* value
Total studies—no./total no. (%)	283/397 (71.2)^a^	108/397 (27.2)^a^	–
Median sample size (IQR)	87 (44-170)	65 (42-106)	.017
Median length of enrollment (IQR)	36 (21-50)	31 (20-46)	.32
Median length of follow-up (IQR)	24 (12-36)	18 (12-31)	.35
Mean number of sites (SD)	6.7 (9.9)	1.9 (4.7)	<.0001
Source of funding—no./total no. (%)			
Institutional	94/283 (33.2)	59/108 (54.6)	<.0001
Industry	95/283 (33.6)	4/108 (3.7)	
Government	58/283 (20.5)	21/108 (19.4)	
Charitable	9/283 (3.2)	2/108 (1.9)	
Unspecified	27/283 (9.5)	22/108 (20.4)	
Subspecialty—no./total no. (%)			
Spine	198/283 (69.9)	76/108 (70.4)	<.0001
Functional	37/283 (13.1)	3/108 (2.8)	
Cerebrovascular	20/283 (7.1)	13/108 (12.0)	
Neurotrauma	3/283 (1.1)	9/108 (8.3)	
General	11/283 (3.9)	1/108 (0.9)	
Neuro-oncology	10/283 (3.5)	3/108 (2.8)	
Pediatric	4/283 (1.4)	3/108 (2.8)	

^a^Does not add up to 397, because 6 studies recruit from both.

## DISCUSSION

We conducted a systematic appraisal of the design characteristics, funding source, and subspecialty of 397 neurosurgical RCTs published between 2003 and 2016 to compare relevant methodological characteristics between trials both led by and including population samples from HICs and LMICs. With 73.3% of studies having been led by HICs, the data clearly present the substantial deficit in terms of high-quality LMIC-led neurosurgical research. In addition, though China is considered an upper-middle-income country, the United States National Science Foundation released a report in early 2018 declaring, for the first time, that China had surpassed the United States as the world's largest producer of scientific articles.^13^ Of the 397 RCTs included in our analysis, 71 were led by China. Whereas 26.7% of studies were led by LMICs, when excluding the 71 studies led by China, only 8.8% were led by LMICs.

### Design Characteristics of HIC and LMIC Neurosurgical RCTs

Although median enrollment and length of follow-up were similar between HICs and LMICs, median sample size and mean number of sites were significantly different. The importance of sample size is critical in research, as one can draw a precise and accurate conclusion only with an appropriate sample size. With less funding and fewer resources, it is unsurprising that studies in LMICs have smaller sample sizes. In a study of 102 RCTs with negative results, Moher et al^14^ found that only 36% had 80% power to detect a relative difference of 50% between 2 groups in a simple 2-group parallel design trial. Therefore, appropriate sample sizes are necessary in order to provide meaningful results.^15^

Furthermore, the mean number of enrollment sites for RCTs led by LMICs was found to be 1.9, compared to 6.7 of those led by HICs. Single-center trials are thought to have limited external validity, as “interventions tested in a single clinical environment are not necessarily generalizable to a broader population.”^16^ Factors such as differences in resources, case-mix, and of end-of-life practices can influence prognosis.^16^ Hence, single-center studies often have limited generalizability. Dechartres et al^17^ found that single-center RCTs showed larger treatment effects than did multicenter RCTs, suggesting that results of single-center trials are frequently contradicted when similar trials are performed in multicenter settings.

### Comparison of Funding Sources in HIC and LMIC Neurosurgical RCTs

The distribution of funding sources between HIC- and LMIC-led studies is likely a major factor in the differences observed in median sample size and mean enrollment sites. Although the majority of funding for HIC-led studies came from industry (33.7%), over half of the 106 LMIC-led studies were institutionally funded (54.7%).

An increase in government investment for local research is thus essential. Although our data suggest that government funding was similar between HICs and LMICs, we again have to take into account China's economic model, in which centralized assessment of needs and governmental funding plays an important role. Thus, whereas 18.9% (n = 20/106) of LMIC-led trials received government funding, 19/20 of those trials were led by China. The one trial that was not led by China was led by a United States investigator based in Uganda, yet, was receiving government funds from the NIH. One could make a strong argument that in fact no LMIC-led trials were government funded.

Industry-funded studies in LMICs comprised 3.8%, with 75% of those having been carried out by China. It is important to note that it is not that industry funding is scarce throughout all global health-related RCTs. In fact, Wong et al^18^ found that 42% of oncology RCTs carried out in LMICs between 1998 and 2008 were industry-funded. However, a few of these trials are simply vertical research projects with HIC researchers focusing on commercial drug and product development, thus limiting the opportunity for research capacity building in LMICs.^19^ In light of greater public awareness of the various ethical issues arising when vertical research projects are taking place in LMICs, many pharmaceutical companies have begun publishing policy positions describing their approach to conducting clinical trials. For example, in May 2019, GlaxoSmithKline (GSK) published a 4-page brief outlining their approach to conducting clinical trials.^20^ In that brief, it is stated that in principle they would “not conduct clinical trials in countries where we do not intend to pursue registration and to make the product available for use.” It is also mentioned that “the type of reimbursement or other compensation offered by GSK to trial participants for their time and/or for any discomfort experienced is appropriate to the local economy and approved by independent ethics committees. Payments to investigators or their institutions reflect fair market value and are in line with local practices.”

Funding bodies, such as the Wellcome Trust in the United Kingdom, have also issued relevant guidance.^21^ The Wellcome guidance states that “investigators conducting Wellcome-funded research involving people living in LMICs must demonstrate that their research is responsive to the healthcare needs or priorities within that country” and that “the outcomes of such research should be likely to lead to relevant and sustainable health benefits to people in those areas where the research is undertaken.”

In many areas where local resources and funding is minimal, collaboration with foreign groups becomes an attractive option. However, relying on international institutional grants limits the researchers’ freedom by potentially favoring donors’ thematic focus.^22^ Changing this would require that LMIC physicians and research leaders advocate the importance of clinical trials for health outcomes and institutional capacity and emphasis to governments and ministries of health that strategic allocation of money can maximally optimize guidance of relevant health policy.

### Comparison of Type of Neurosurgical Intervention

Every year, there are approximately 13.8 million new operative cases performed for neurosurgical disease, without including degenerative spine disease and osteoporotic vertebral fractures. The number of patients who would end up severely disabled or dead without surgical treatment for these conditions cannot be estimated easily, especially in LMICs. Traumatic brain injury (TBI) and stroke-related conditions constitute roughly 60% of cranial essential neurosurgical volume, with tumors, hydrocephalus, epilepsy, and infectious-related conditions accounting for the majority of the remaining proportion of disease.^23^ Yet, whereas 60% of operations requiring neurosurgical care are TBI or stroke-related, only 7.5% and 11.3% of neurosurgical RCTs in LMICs evaluated interventions categorized as neurotrauma and cerebrovascular, respectively.

In the Executive Summary of the Global Neurosurgery Initiative at the Program in Global Surgery and Social Change, Dewan et al^23^ make the argument that although degenerative spine disease, chronic pain, movement disorders, and many other conditions are well known to cause disability and suffering if left untreated, these conditions, most notably degenerative spine disease, do not currently qualify for inclusion as essential, lifesaving, neurosurgical interventions. Therefore, it is surprising that more than two-thirds of RCTs in LMICs are devoted towards spine surgery.

A total of 69 million individuals are estimated to suffer TBI from all causes each year. The vast majority of this burden affects populations in LMICs, where 85% of the world's population live. The healthcare systems in LMICs encounter nearly 3 times as many total TBIs than those in HICs. Unfortunately, limited data suggest that LMIC patients have over twice the odds of dying following severe TBI in addition to a greater degree of disability.^24,25^ This makes it increasingly important that adequate research funding, time, and policy proposals are appropriately allocated to essential neurosurgical care. It is not enough to rely on neurotrauma research from HICs and extrapolate results to develop guidelines in an LMIC. Chestnut et al^26^ note that although pathophysiology of TBI is similar in HICs and LMICs, there are “important differences in demographics and injury mechanism that may influence outcome.” Furthermore, specific intracranial injuries identified on computed tomography differ significantly and the care they receive may be substantially different when compared to HICs.^27^

### Neurosurgery Research in a Global Context

The importance of LMIC research capacity is not a novel idea; the 1990 Commission on Health Research for Development stated that strengthening research capacity in LMICs is “one of the most powerful, cost-effective, and sustainable means of advancing health and development.”^19^ The 2013 World Health Report stated that all “nations should be producers and users of research as well as consumers,” emphasizing that this was not yet the case.^28^ Thus, as important as it is to advocate for building and improving neurosurgical research capacity in LMICs, it is more important to advocate for establishing centers of excellence for the development of long-term capacity to conduct advanced research in multiple areas of medicine. These centers of excellence should be based on equitable partnerships and should be driven by LMIC needs.^29^

It would be the duty of these “centers of excellence” to set local research priorities based on local unmet needs. With limited resources, a lack of prioritization risks selection of research that is either most closely aligned with current activity or research that is easier to implement.^30^ We argue that research priority setting will help direct limited resources to areas of greatest need and impact. To realize this vision, communication between research institutions, funding bodies, and policy makers must improve. Research agendas with concrete strategies, time-dependent targets, and requirements for outcome reporting and implementation must be developed and built into the National Surgical, Obstetric and Anesthesia Plans for each country. This is a national health plan specifically focused on surgical capacity and access in a country. Without these assessments, the quality and impact of research capacity building will stall, and with it the barrier between disease and health.

Granted, it was not until 2015 when *The Lancet Commission on Global Surgery* was published that the global health community was confronted with new data that presented the global burden of disease commensurate with a lack of resources invested towards providing safe and affordable access to surgery and anesthesia. This is not a justification of the scarcity of trials in or led by LMICs, but a major step in the right direction. Another major step towards improving neurosurgical research capacity in LMICs was the publication of *The Lancet Neurology Commission on Traumatic Brain Injury: Integrated Approaches to Improve Prevention, Clinical Care, and Research* late in 2017, which identified high rates of neurotrauma in LMICs as a key agenda item in line with the UN Sustainable Development Goals (SDGs). SDG 3.6, in particular, calls for halving global deaths and injuries from road traffic accidents. With 90% of TBI-related deaths occurring in LMICs, a concerted effort is needed to reduce the 10/90 gap, whereby less than 10% of current global research funding goes towards diseases that afflict more than 90% of the population.^31,32^ The Commission notes, “[TBI] studies in LMICs are urgently needed,” given the difference and challenges of TBI care in LMICs when compared to HICs. The report continues by recommending that solutions for improving TBI care and outcomes in LMICs should be tailored to local needs and resource availability rather than replicating strategies in HICs.^33^

**FIGURE 2.**
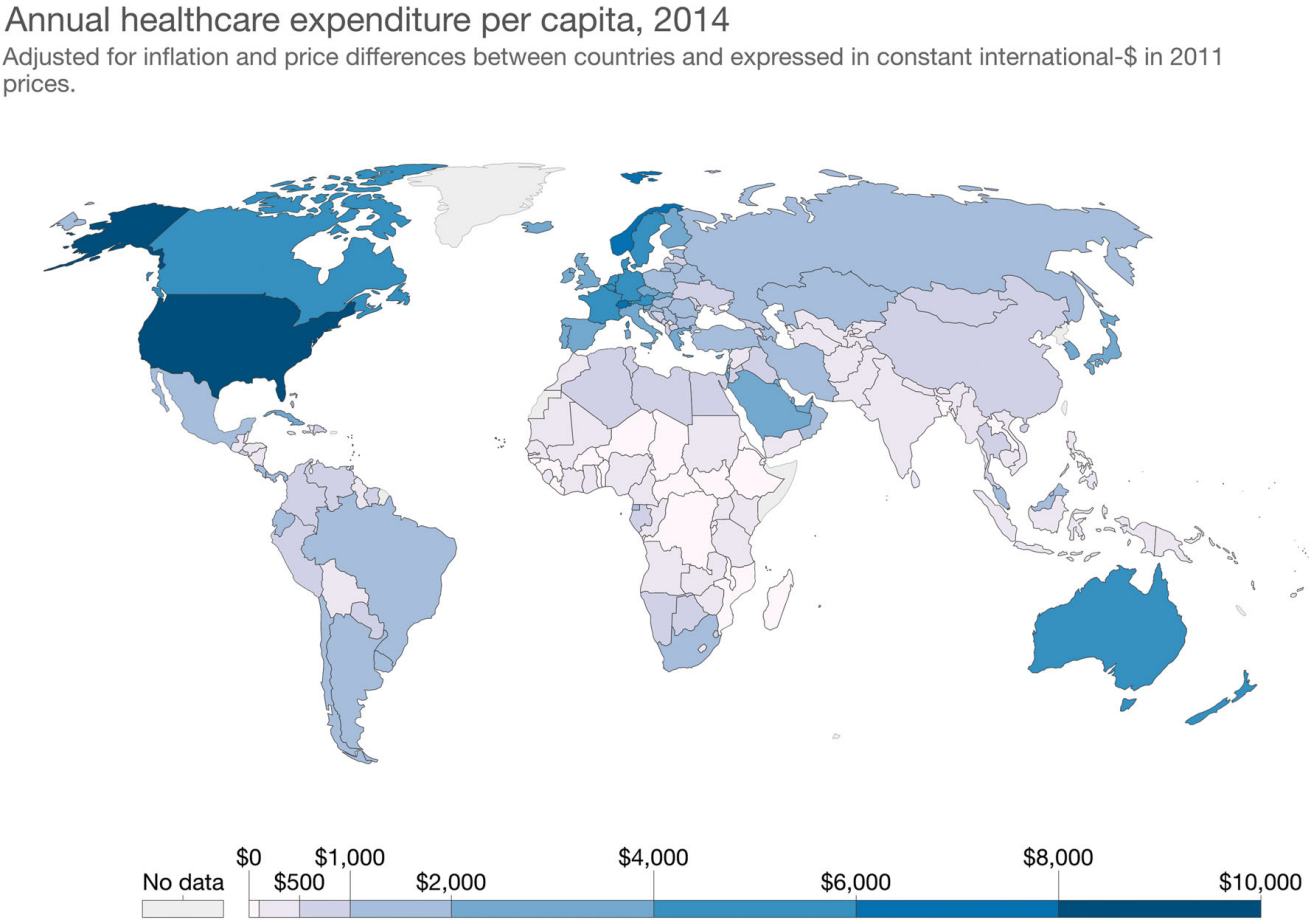
The annual healthcare expenditure per capita ranges from 24.96 international-$ in the Central African Republic to 9402 international-$ in the United States, based on data from fiscal year 2013/2014. Total health expenditure is the sum of public and private health expenditures as a ratio of total population. Data are in international dollars converted using 2011 purchasing power parity (PPP) rates. “Annual healthcare expenditure per capita, 2014” map from “Financing Healthcare” by Estaban Ortiz-Ospina and Max Roser (https://ourworldindata.org/financing-healthcare), licensed under CC BY 4.0 (https://creativecommons.org/licenses/by/4.0/deed.en_US). Data are from World Bank–World Development Indicators, World Health Organization Global Health Expenditure database (see http://apps.who.int/nha/database for the most recent updates).

The distribution of neurosurgical RCTs (Figure 1) is unsurprising given the robust programs for financing such RCTs. The National Institutes of Health invests nearly 37.3 billion annually in medical research for the American people and the United States allocates 18% of its gross domestic product (GDP) towards total healthcare expenditure.^34^ Most Sub-Saharan African countries allocate less than 5% of their GDP towards healthcare, with many countries in South Asia allocating less than 3% (Figure 2).^35^ Although a country's allocation of GDP towards healthcare is not the same as research funding for neurosurgical RCTs, it is helpful to show how countries with the highest percentage of GDP allocated towards healthcare also lead the most neurosurgical RCTs.

LMIC governments should be encouraged to support research, as the majority of LMIC-led trials are supported by “institutional funds.” Overall, neurosurgical RCTs are being led by investigators in a small number of well-funded, urban environments. More must be done to incentivize LMIC governments to invest in resources to help train investigators capable of carrying out robust RCTs. Economic data on how untreated neurosurgical disease affects national GDP and how investing in more trials centered around TBI and stroke-related disease will lead to a decrease in disability-adjusted life years (a measure of overall disease burden, expressed as the number of years lost because of ill health, disability, or early death) and thus help cushion against the enormous economic loss. There is a lack of trauma-related evidence from LMICs, and policy makers have advocated for a standardized approach to economic evaluation of injury in LMICs to prioritize investing in interventions capable of preventing injury.^36^

In our view, neurosurgeons and researchers from HICs who are interested in advancing global neurosurgery should aim to develop equitable partnerships with neurosurgeons and researchers from LMICs. Policy makers, funding agencies, and universities, all working together, are essential to building LMIC-led, sustainable research capacity. In 2017, the United Kingdom department of health allocated more than $200 million to stimulate research that would directly benefit patients in LMICs. Of that, more than 2.3 million was awarded to establish the NIHR Global Health Research Group on Neurotrauma. The Group is composed of clinicians and researchers from 11 LMICs and 3 HICs with the aim of creating centers of excellence with research programs directly focused on improving disparities in neurosurgical care. To help facilitate the dissemination of findings, the Group has partnered with the British Medical Journal Research to Publication programme^37^ in addition to providing online education in methods of research for all participating institutions.

## CONCLUSION

We have established that there is a substantial disparity between HICs and LMICs in the number of published neurosurgical RCTs. We have also shown that nearly 75% of all neurosurgical RCTs were led by HICs. Excluding China, only 8% of neurosurgical RCTs were led by LMICs. Studies led by LMICs were smaller, with both fewer enrollment sites and enrollees. Furthermore, although industry funding was the major funding source for HIC-led studies, only 4% of studies in LMICs were industry funded. Only 7.5% and 11.3% of neurosurgical RCTs in LMICs evaluated interventions categorized as neurotrauma or cerebrovascular, respectively, even though these conditions cause far more neurological disability. A concerted effort to invest in research capacity building in LMICs is an essential step towards ensuring context- and resource-specific high-quality evidence is generated.

### Disclosures

This work is supported by the NIHR Global Health Research Group on Neurotrauma, which was commissioned by the National Institute for Health Research (NIHR) using United Kingdom aid from the United Kingdom Government (project 16/137/105). The authors have no personal, financial, or institutional interest in any of the drugs, materials, or devices described in this article. Dr Hutchinson is the Chief Investigator of the RESCUEicp and RESCUE-ASDH randomized trials. Dr Servadei has received personal fees from Takeda Pharmaceutical Company, grants and personal fees from Integra LifeSciences, and grants and personal fees from Finceramica SpA. Mr Griswold was supported by the Stanford University Medical Scholars Program and the Gates Cambridge Trust. Dr Hutchinson was supported by a Research Professorship from the NIHR, the NIHR Cambridge Biomedical Research Centre, a European Union Seventh Framework Programme grant (CENTER-TBI; grant no. 602,150), and the Royal College of Surgeons of England. Dr Kolias was supported by a Clinical Lectureship from the School of Clinical Medicine, University of Cambridge, and the Royal College of Surgeons of England.
